# Vaginal birth after cesarean section: A quantitative study exploring women’s understanding and experience regarding VBAC rates in Greece

**DOI:** 10.18332/ejm/168253

**Published:** 2023-07-24

**Authors:** Stefania Moysiadou

**Affiliations:** 1 International Hellenic University, Thessaloniki, Greece

**Keywords:** cesarean section, cesarean section vaginal birth after cesarean delivery, VBAC, maternal health services, patient satisfaction, Greece

## Abstract

**INTRODUCTION:**

Vaginal birth after cesarean section (VBAC) is a choice of birth that provides many advantages for women. This study presents women’s VBAC experience in a Greek population. The study’s aims were mainly to determine the reasons for choosing VBAC, women’s feelings during pregnancy and their experience, and level of post-birth satisfaction.

**METHODS:**

This study is sampling research which has a cross-section retrospect study design. The study was held via the internet due to a self-administered questionnaire which is comprises both open-ended and close-ended questions. Data analysis was performed in S.P.S.S. 20 and Microsoft Excel.

**RESULTS:**

A total number of 473 women participated in this study. The findings showed that during pregnancy and childbirth over 50% of women felt very happy and satisfied, while 35% to 40% felt moderate or no fear at all. Furthermore, 96.48% of them would attempt for a VBAC birth again while 97.36% would recommend this way of delivery to other women. There was a total of 78.85% of succeeded VBAC. The main reason for women to choose VBAC for a birth option were the desire for a normal birth (23.1%), the thought of vaginal birth as the normal way of giving birth (22.4%), to avoid another surgery (14.2%) and to experience a vaginal birth (10%).

**CONCLUSIONS:**

VBAC is an option that needs to be offered more in Greece, and needs improvement in obtaining informed consent in obstetric care services. More studies are required to draw further conclusions.

## INTRODUCTION

Cesarean section (CS) rates rose in the USA from 5% in 1970 to 32% in 2016^1^, in Europe from 11% in 1990 to 25% in 2014, and in Asia from 4% to 19%^2^. More recent data, according to WHO, shows that CS rates are up to 34% in the USA, 24% in the UK, 27% in China and 50% in Brazil^3^. Regarding Greece, according to a study which was held in the 1^st^ Department of Obstetrics and Gynecology of the Aristotle University of Thessaloniki at the Hippokrateio General Hospital in Thessaloniki, Greece, the rates have increased from 13% in 1977 to 29% in 2000^4^. The latest reports show a further increase to 56% in 2017 in Greece^5^. It should be emphasized that the World Health Organization (WHO) recommends the CS rates to be between 10% and 15%^6^.

In 1985 VBAC rates in the USA were 5% and increased to 28% in 1996, but due to the increased number of complications related to VBAC and other contributing factors, VBAC rates had decreased to 8% by 2006^1^. In the period from 2016 to 2018, the VABAC rates in the USA rose up 12% to 13%^7^. This rate was up to 38–55% in Finland, the Netherland and Norway and up to 29–36% in Germany, Ireland and Italy^8,9^.

The factors that influence women to choose a VBAC birth over a repeated CS are the feeling women have of having failed for not having given a vaginal birth in their previous pregnancy or the feeling of having missed the experience of a vaginal birth, the experience they had during their previous birth, and the recovery they had from their previous CS^10-12^. The information they received from their healthcare professional (HCP) and their acquaintances as well as the advantages and disadvantages and the risks and safety of each mode of birth (VBAC or repeated CS) influenced women when making a decision of the mode of birth^10-12^.

Furthermore, there have been recommendations from the Royal College of Obstetricians and Gynaecologists (RCOG), the American College of Obstetricians and Gynecologists, the Society of Obstetricians and Gynecologists of Canada (SOGC) as well as from the Hellenic Society of Obstetrics and Gynecology (HSOC) presenting the indications for a safe VBAC. According to these recommendations women who have had a previous lower segment cesarean delivery should be consulted about the mode of birth they can have in a next pregnancy^1,13-15^. Women who attempt a VBAC must have a singleton pregnancy, cephalic presentation, and single previous lower segment cesarean delivery^1,13,15^. VBAC can be considered as an option of birth for women who have had two cesarean sections or more^1,13-15^. Contraindications for attempting a VBAC are previous or suspected classical CS^1,14,15^, previous inverted T or low vertical uterine incision^1,13,15^, previous uterine rupture^1,13-15^, previous major uterine reconstruction^1,15^, woman requests ERCS rather than a VBAC^15^, more than 3 previous CSs^13^, and women who have other contraindications to vaginal birth^1,14^. Induction of labor and augmentation is not contraindicated for women attempting VBAC, but women should be informed that they increase the risk of uterine rupture^1,13-15^. VBAC should take place in a suitably staffed and equipped hospital, where emergency CS and neonatal resuscitation can be performed immediately^1,13,14^. The fetal heartrate should be continuously monitored^1,13-15^. Analgesia during VBAC is not contraindicated^1,13-15^.

Since there have not been many VBAC related studies in Greece, the aim of this study was mainly to determine the reasons behind the decision for a VBAC, women’s feelings during the pregnancy and their experience, and level of post-birth satisfaction. The reasons for undertaking this study were to explore the family environment’s support of women’s decision to attempt a VBAC and if there was support from their partner with this decision, as well as if their presence and that of the midwife during childbirth helped the women, and whether the HCPs were supportive regarding VBAC as a choice of birth or not, and to analyze the type of information that women receive about VBAC. Finally, the study aims to collect data on the reasons for the previous CS of women, as well as on the outcome of their subsequent childbirth.

## METHODS

### Study design, setting and participants

This study is of cross-sectional study design, with non-random sampling, as it corresponds to a specific group of women in a specific time period^16-18^. The study took place on Greek social media, from July 2018 to February 2019. The questionnaire target sample consisted of women who had previously given birth by cesarean section and then attempted to give birth by vaginal delivery in Greece. Women’s age, gestation weeks as well as the number of previous CSs were not taken into account as inclusion criteria. The timeframe of the VBAC birth was not specified. The only inclusion criterion that was taken into account for this study was participants lived in Greece, as it was considered that they were living in Greece during the VBAC.

### Study instrument

A structured electronic questionnaire was developed. A questionnaire on Google forms was used to conduct the study. It contained close-ended and open-ended questions as well as Likert scales which were used for the participants to rate the scale of the emotions they felt, through particular types of questions^16^. The total number of questions (including the sub-questions) in the questionnaire was 47. The first questions referred to demographic data followed by questions about the previous cesarean section. There were questions also regarding the information women received and their HCP. Questions followed about the reasons for choosing VBAC as a mode of birth and the support women received from their family environment during pregnancy and childbirth. The last two groups of questions referred to the time of birth and the newborn. Confidentiality was ensured by keeping the participation anonymous, the participation was voluntary, and women could withdraw at any point. The main limitation of the questionnaire is that the participant’s residence is not specified during the VBAC birth, as there was no question for it. In addition, even though women’s age was not taken into account, the participants had to choose from 20 years of age to 50 years, which led women aged <20 years or >50 years to be excluded.

### Data collection

The electronic questionnaire that was constructed for this study was first published on July 2018 on various groups on social media and more specific on Facebook. The questionnaire was published on each group accompanied by a text which explained to the group members that this questionnaire referred to women who have had a previous cesarean section and were trying a VBAC in Greece. Permission for publishing the questionnaire on these various groups was taken from the group’s administrator before the post was published. The questionnaire was reposted on these groups several times with a time gap of one to three months, accompanied always by the same text. The context of these groups was about VBAC, maternity, midwifery and gynecology. It was available for access until February 2019. The participants’ answers were automatically saved at the platform of Google Forms.

### Statistical analysis

Statistical processing was performed with the Statistical Package S.P.S.S. 20 and Microsoft Excel. For the analysis and extraction of statistically significant inductive conclusions, Pearson’s χ^2^ and t-tests were used^19-22^.

## RESULTS

This questionnaire was available online from 11 July 2018 to 19 February 2019. The number of participants in this study was 473, of whom 19 were excluded for not meeting the inclusion criteria, resulting in a total of 454 participants. Table 1 presents the participants’ demographic characteristics. Most (70%) of the participants were aged 31–40 years. Nonetheless, it seems that some CS were not indicated.

**Table 1:** Participants’ demographic characteristics, Greece, 2018–2019 (N=454)

*Characteristics*	*n*	*%*
**Age** (years)		
20–30	110	24.2
31–40	318	70.0
41–50	26	5.7
**Residence**		
Athens	193	42.5
Greece	1	0.2
Thessaloniki	85	18.7
**Prefecture**		
Etoloakarnania	5	1.1
Argolida	1	0.2
Arkadia	2	0.4
Arta	1	0.2
Achaia	10	2.2
Boeotia	2	0.4
Chalkidiki	3	0.7
Chania	14	3.1
Corfu	3	0.7
Cyclades	6	1.3
Drama	3	0.7
Dodekanisa	9	2.0
Evros	1	0.2
Evia	10	2.2
Fthiotida	3	0.7
Florina	1	0.2
Heraklion	9	2.0
Ileia	2	0.4
Imathia	7	1.5
Ioannina	9	2.0
Kavala	4	0.9
Karditsa	1	0.2
Kefallinia	1	0.2
Kozani	4	0.9
Korinthias	4	0.9
Laconia	1	0.2
Larisa	3	0.7
Lasithi	2	0.4
Lefkada	4	0.9
Magnisia	16	3.5
Pella	2	0.4
Pieria	7	1.5
Preveza	1	0.2
Rethymnon	4	0.9
Rodopi	5	1.1
Samos	2	0.4
Serres	5	1.1
Trikala	3	0.7
Xanthi	3	0.7
Zakynthos	2	0.4
**Education level**		
High school graduate	49	10.8
ΙΕΚ graduate	70	15.4
TEI-HEI graduate	199	43.8
Postgraduate/doctoral degrees	123	27.1
Other	13	2.9
**Employed**		
Yes	301	66.3
No	153	33.7

IEK: Institute of Vocational Training. ΤΕΙ: Technical Education Institute. Higher Education Institute.

**Figure 1:**
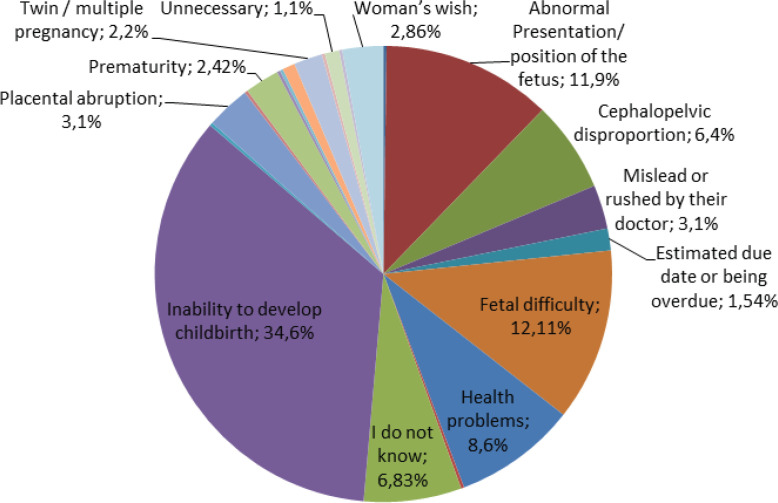
Most common reasons for the previous cesarean section

As shown in Figure 1, the most common reasons for the previous cesarean section (CS) were failure of progress (34.6%) , fetal distress (12.11%), abnormal presentation/position of the fetus (11.9%), other medical complexities (8.6%), and cephalopelvic disproportion (6.4%). Additionally, 6.83% stated they were not aware of the rationale for their previous CS, and 3.1% reported feeling misled or rushed by their doctor.

When participants were asked where they first heard about the possibility of a vaginal birth after cesarean (VBAC), 71.6% stated from the media and the internet, 15.2% from friends and relatives, 7% from their obstetrician, and 6.2% from their midwife. Furthermore, over two-thirds of women were informed about the option of VBAC prior to their next pregnancy. According to women, the information they received about VBAC contained success, failure, and complications rates for VBAC and repeated CS at a rate of 60%, in contrast to 23.8% of women who stated that it did not contain such information, while 10.4% did not remember, and 6.4% would have liked more detailed information. When asked if this kind of information would scare them, 77.8% of them said they were not afraid at all, 13.4% felt a little fear, 7.3% moderate fear, and only 1.5% felt very much fear. When asked to comment on the information they received, a small group of women (65 in total) made comments, and the majority of them stated that they had received full information or good but incomplete information.

According to the participants, one-third of their family environment was moderately positive, while almost another one-third was minimally positive, as well as very positive about their decision of VBAC. Referring to the support they received from their partner, 52% stated their partner was very much supportive of their decision to have a VBAC, while a further 19.2% were very supportive. Healthcare professionals (HCP) refused to offer them the opportunity to have a VBAC in 51% of the cases. Regarding HCPs who were open to facilitate VBAC, 65.6% stated they had to look for one, while 6.4% stated they did not have to find one, and 28% did not respond. When asked about the difficulty of finding an HCP who supported VBAC as a birth option, it was not difficult to find one at a rate of 50%, while 24.7% had difficulty identifying an assisting HCP, and 28.2% did not respond.

Table 2 (n=450) shows all the reasons the participants stated for choosing VBAC as a mode of birth. The majority of them chose VBAC because they wanted to give birth naturally, felt that vaginal delivery was the normal way to give birth, wanted to avoid one more surgery, and desired to have a birth experience. They considered VBAC to be the best choice for them and wanted to avoid another surgery.

**Table 2:** Reasons for choosing VBAC as a birth option, Greece 2018–2019 (N=450)

*Reasons*	*n*	*%*
Absence of cause for cesarean section	4	0.89
Avoiding extra surgery	64	14.22
Benefits of normal childbirth	26	5.78
Best option	41	9.11
Better consolidation of breastfeeding	5	1.11
Better recovery	28	6.22
Cesarean section only when needed	10	2.22
Childbirth in the past with normal birth/VBAC	8	1.78
Childbirth when it is time	21	4.67
Confidence for a successful VBAC	5	1.11
Denial of ‘once cesarean section always cesarean section’	2	0.44
Direct contact with newborn	3	0.67
Desire for normal birth	104	23.11
Desire for many children	5	1.11
Eligibility for VBAC	2	0.44
Emotional reasons	12	2.67
Encouragement of doctor for VBAC	3	0.67
Experience	45	10.00
Fear of cesarean section of its complications	20	4.44
Feeling worth it	1	0.22
Freedom of movement	1	0.22
Good example	1	0.22
Health for woman and newborn	19	4.22
Healthy pregnancy	10	2.22
Less complications	10	2.22
Mandatory	2	0.44
More active role in childbirth	2	0.44
Normal way of childbirth	101	22.44
Out of stubbornness	6	1.33
Personal reasons	2	0.44
Presence of husband	3	0.67
Primary choice for pregnancy after cesarean section	1	0.22
Psychological reasons	24	5.33
Random	1	0.22
Right to a normal birth	10	2.22
Respect for the woman/newborn	10	2.22
Safer/more trustworthy	16	3.56
Traumatic experience of previous cesarean section	28	6.22
Unnecessary previous cesarean section	15	3.33

Table 3 shows how women felt during their pregnancy about their choice of VBAC. One-third stated that they felt little to no fear, while others reported feeling anxious or having minimal anguish. Half of the participants expressed having no doubts at all, and more than half of the candidates reported being very happy and satisfied with their decision to choose VBAC as a birth option. Women who gave birth vaginally felt happier and more satisfied with their choice of VBAC during pregnancy compared to those who had a cesarean section (Monte Carlo simulation, p<0.0004; Crammer’s V=0.193, p<0.0004, and Monte Carlo simulation, p<0.0004; Crammer’s V=0.292, p<0.0004, respectively).

**Table 3:** Feeling about the choice of a VBAC during pregnancy and childbirth, Greece 2018–2019 (N=454)

	*VBAC birth during pregnancy*		*Feelings during VBAC childbirth*	
	*n*	*%*	*n*	*%*
**Fear**				
Not at all	177	39.0	194	42.7
Minimum	156	34.4	137	30.2
Moderate	93	20.5	87	19.2
Very	23	5.1	22	4.8
Very much	5	1.1	14	3.1
**Anxiety**				
Not at all			136	30.0
Minimum	158	34.8	133	29.3
Moderate	114	25.1	113	24.9
Very	67	14.8	50	11.0
Very much	12	2.6	22	4.8
**Anguish**				
Not at all	62	13.7	84	18.5
Minimum	122	26.9	119	26.2
Moderate	126	27.8	119	26.2
Very	112	24.7	87	19.2
Very much	32	7.0	45	9.9
**Doubt**				
Not at all	221	48.7	242	53.3
Minimum	120	26.4	111	24.4
Moderate	83	18.3	61	13.4
Very	22	4.8	29	6.4
Very much	8	1.8	11	2.4
**Happiness**				
Not at all	3	0.7	19	4.2
Minimum	8	1.8	9	2.0
Moderate	37	8.1	39	8.6
Very	147	32.4	97	21.4
Very much	259	57.0	290	63.9
Total	454	100	454	100
**Satisfaction**				
Not at all	8	1.8	19	4.2
Minimum	6	1.3	16	3.5
Moderate	39	8.6	45	9.9
Very	123	27.1	73	16.1
Very much	278	61.2	301	66.3

Regarding negative feelings about their choice of VBAC during pregnancy, such as fear, anxiety, and doubt, the distribution did not show statistically significant differences based on the way they gave birth (Fear: Monte Carlo simulation, p=0.177; Anxiety: χ^2^=7.898, df=8, p=0.444, Anxiety: χ^2^=11.630, df=8, p=0.169; Doubt: χ^2^=10.386, df=2, p=0.239).

Table 3 also shows how women felt about their decision to choose VBAC as a birth option during childbirth. Almost half of the women felt no fear at all (42.7%). Referring to stress levels, one-third stated that they did not feel any stress or felt minimal stress. The majority of women reported a low level of anguish, while over 50% expressed no doubt, feeling very happy, and being highly satisfied with their choice of a VBAC birth during childbirth.

Regarding how women felt during VBAC childbirth, no statistically significant differences in fear were observed based on the way women gave birth (successful VBAC or CS) (p<0.05, Crammer’s V=0.154 <0.19). However, those who gave birth by CS felt more anxious than those who gave birth vaginally (χ^2^=29990, df=8, p<0.0004; Crammer’s V=0.182, p<0.0004). Similar findings were observed regarding the intensity of anguish (χ^2^= 34774, df=8, p<0.0004; Crammer’s V=0.196, p<0.0004). Women who gave birth by CS also felt more doubt (χ^2^=78143, df=8, p<0.000; Crammer’s V=0.293, p<0.000). On the other hand, those who gave birth vaginally felt happier during VBAC birth (χ^2^=109424, df=8, p<0.000; Crammer’s V=0.347, p<0.000), and reported higher levels of satisfaction during VBAC childbirth (χ^2^=170805, df=8, p<0.000; Crammer’s V=0.434, p<0.000). Finally, no statistically significant difference was found in the distribution of women who had an instrumental birth (vacuum/forceps).

When women were asked if they had regretted their choice of VBAC when the time to give birth had come, the majority of them stated that they had no regrets (97.6%) and did not regret their decision to have a VBAC birth. A minority of 2.4% (n=11) mentioned that they had regretted it. When asked for the reasons, they stated that VBAC would be dangerous, time-consuming, and mentioned the pain experienced during birth. Additionally, pressure from doctors to have a cesarean section during VBAC or the admission of the newborn to the neonatal intensive care unit (NICU) were also reasons for regretting their choice of a VBAC birth.

Regarding women’s experience, 48% reported being completely happy with the VBAC experience, while 33.3% were very happy, 14.3% were moderately happy, and 4.4% were not happy at all. The women who gave birth vaginally expressed significantly higher levels of satisfaction with the VBAC experience (χ^2^=190645, df=6, p<0.0004; Crammer’s V=0.458, p<0.0004).

When asked to explain why they felt the way they did, 13.4% stated that it was a pleasant experience, 6.2% were not happy about their experience because they had a cesarean section, and 3.7% felt that the interventions of healthcare professionals during childbirth were not pleasant for them. Approximately 69.4% of participants did not provide an answer.

In 80.2% of the cases, the partner was present during the VBAC, while in 19.8% of the cases, the partner was not present. Statistically, more women who gave birth vaginally had their partner present during birth (χ^2^=29924, df=2, p<0.000; Crammer’s V=0.257, p<0.000). When asked if the presence of their partner was helpful during childbirth, 85.9% stated that it was helpful, while 14.1% stated that their partner’s presence did not help them during childbirth.

According to the women, the presence and support of the midwife during VBAC were very important to them (Table 4); 67.4% of women were familiar with the midwife, while 24.23% did not have a familiar midwife during childbirth, and 8.4% saw their midwife for the first time during childbirth. Women who had a familiar midwife present during childbirth considered the midwife’s presence and support to be statistically significant and quite strong, more so than those who did not have a familiar midwife present (χ^2^=128833, df=8, p<0.000; Crammer’s V=0.377, p<0.000).

**Table 4:** Importance of presence: support of the midwife during VBAC childbirth, Greece 2018–2019 (N=454)

*Important*	*n*	*%*
Not at all	24	5.3
Minimum	17	3.7
Moderate	43	9.5
Very	100	22.0
Very much	270	59.5

Regarding a subsequent pregnancy, 96.5% of the participants stated that they would try a VBAC again, while 3.5% would not. When asked if they would change anything in the next VBAC (n=252), 32.5% stated that they would not change anything, 17% mentioned they would prefer less frequent vaginal examinations, 13.1% expressed a preference for a home birth, 8.3% would change their way of thinking about birth, including not choosing epidural anesthesia as pain relief, and 7.5% would choose another healthcare professional. The majority of participants (97.4%) would recommend VBAC to other women, and a majority (95.6%) also believed that VBAC should be offered to all women with a previous cesarean section who do not have a medical indication for a repeat cesarean section.

In this study, the success rate of vaginal birth was 62.8 %, while 16.1% had an instrumental birth (vacuum/forceps), and 21.1% had a cesarean section. The majority of women (82.4%) experienced spontaneous onset of labor, while 17.6% had induced labor. When asked if there were any complications, 19.6% answered yes, 80.0% answered no, and only two women (0.4%) did not provide a specific answer.

## DISCUSSION

In the present study, as well as in the studies of Qazi et al.^23^, Nousia et al.^24^, and Mone et al.^25^, the success rate of vaginal delivery exceeds 60%. The majority did not report complications. In relation to the main causes of a previous CS, they were consistent with the studies of Qazi et al.^23^ and Nousia et al.^24^, which identified failure to progress, fetal distress, and abnormal fetal position/presentation. In relation to the bibliography, failure to progress in the first or second stage, fetal distress during labor, abnormal presentation/position of the fetus (hip projection, transverse shape, etc.), cephalopelvic disproportion, abnormalities of the placenta, and other maternal complexities are indications for a CS^26-28^. According to these statements, we can conclude that most previous CS were performed while meeting the conditions for a CS. Nonetheless, it is clear that some CS were not indicated.

Regarding the information offered or received, in this study, women were initially informed about VBAC as an option through the media and the internet. However, two-thirds of the women felt that the information received from healthcare providers (HCPs) was appropriate. In the study of Davis et al.^12^, women also reported feeling well informed by their HCPs. In the study of Chen et al.^10^, women received information about VBAC from the internet, acquaintances, and recommendations of obstetricians. They did not receive written information about VBAC from obstetricians and nurses^10^. Studies by Nilsson et al.^9,29^ indicated that women desired to receive correct and comprehensive information about VBAC. According to the World Health Organization (WHO), healthcare professionals play a critical role in improving access and quality healthcare for the population^30^. HCPs provide essential services to promote health, prevent diseases, and deliver healthcare based on the primary healthcare approach^21^. Therefore, it is important for women to receive appropriate and timely information from HCPs, either immediately after a cesarean section or early in their pregnancy, regarding the mode of birth after a cesarean section. The information should include the benefits and risks of both types of birth. This can serve as a means to reduce cesarean section rates^1,9,13,14,31-33^.

Comparing the outcomes of this study with the research of Hollander et al.^34^, it is evident that women received more support from their partners than from their families regarding the decision to have a VBAC. Additionally, in this study, the majority of women had positive emotions regarding the support and presence of healthcare providers, especially midwives, during childbirth. This sentiment was more pronounced among women who had a familiar midwife. The Nilsson et al.^9,29^ and Foureur et al.^35^ studies also highlight the importance of receiving professional support from healthcare providers who are confident, respectful of women’s needs, and inspire confidence in order to improve VBAC rates. The Lundgren et al.^31^ study emphasizes the need for women to be motivated and aware of their options regarding VBAC. It is noteworthy that in this study, half of the participants reported that healthcare professionals who monitored them during pregnancy either refused to offer them the possibility of VBAC or were less supportive compared to midwives. This finding aligns with other studies^8,35,36^.

Based on the data analysis in this study, women who had a vaginal birth during their VBAC reported more positive emotions and higher satisfaction with their experience compared to those who gave birth by cesarean section. This trend is consistent with the findings in the studies of Chigbu et al.^37^, Nilsson et al.^29^, and Davis et al.^12^. Women who did not have a previous vaginal birth and did not achieve a successful vaginal birth in their last pregnancy often perceive it as a sense of failure. Regardless of the outcome of the VBAC trial, most women express their willingness to attempt a vaginal birth in subsequent pregnancies. Moreover, control over decisions related to pregnancy and childbirth, confidence, a good relationship with healthcare providers, and an active labor are important factors for women to have a positive experience during their VBAC trial, as indicated by the studies of Chigbu et al.^37^ and Keedle^8,36^.

Comparing this study with the research of Chen et al.^10^, Attanasio et al.^11^, and Davis et al.^12^, it can be concluded that the main reasons women choose VBAC are to experience a vaginal birth, perceive it as the natural way of giving birth, avoid another surgery, and expect a faster and easier recovery. Additionally, women opt for VBAC to avoid a negative experience similar to their previous cesarean section^10-12^.

### Strengths and limitations

The main limitation of this study is that the survey was conducted through internet forums, which makes it difficult to confirm the validity of all the data provided by the participants. However, this approach was chosen as it allowed for the collection of a large amount of data from diverse locations in Greece within a short period and at no financial cost. In terms of strengths, this study offers a unique opportunity and baseline for understanding the availability and experiences of VBAC in Greece. The findings also emphasize the importance of following global guidelines and making VBAC available to all women in all maternity hospitals. Furthermore, the study suggests the need for further research to gather additional evidence and draw more comprehensive conclusions.

## CONCLUSIONS

This study demonstrates the existence of VBAC as a birth option in Greece and indicates that many women have positive experiences during pregnancy and childbirth. However, it also highlights the need for healthcare professionals to provide VBAC as a viable option and suggests areas for improvement in offering this choice to women. Further studies are recommended to deepen the understanding of VBAC and its implementation in Greece.

## Data Availability

The data supporting this research are available from the author on reasonable request.
